# Assessment of keloids recurrence rate in patients treated by brachytherapy versus electron beam radiotherapy versus surgical excision

**DOI:** 10.1016/j.jpra.2026.02.010

**Published:** 2026-02-21

**Authors:** Nicola Zingaretti, Francesco Paolo Paduano, Francesco De Francesco, Giamaica Conti, Glenda Giorgia Caputo Ammendola, Valeriano Vinci, Francesco Maria Klinger, Pier Camillo Parodi

**Affiliations:** a University of Udine, University Hospital of Udine, Piazzale Santa Maria della Misericordia, 15, Udine, Italy; b AOU Ospedali Riuniti di Ancona, Via Conca 71, Torrette, Italy; c University of Verona, Department of Neuroscience Biomedicine and Movement, Piazzale Ludovico Antonio Scuro 10, Verona, Italy; d IRCCS Humanitas Research Hospital, Via Alessandro Manzoni, 56, Rozzano, Italy; e Azienda Ospedaliera San Paolo-Polo Universitario, Via Antonio di Rudinì, 8, Milano, Italy

**Keywords:** Keloids, Hypertrophic scars, Radiotherapy, Electron beam therapy, Brachytherapy, Surgery

## Abstract

**Background:**

Keloidal scars are benign lesions, and treatment is primarily directed toward symptom relief and cosmetic implications. Given the risk factors, clinical appearance and high recurrence rate, there is no universally accepted treatment. Currently, radiotherapy after surgical excision is recognized as the most efficacious modality. The aim was to compare the efficacy, by means of recurrence rates, between surgical excision alone, electron beam radiotherapy performed after surgical excision, and brachytherapy performed after surgical excision.

**Material:**

This retrospective observational study included 27 patients with overall 48 keloids. Patients were divided into three groups: Group 1 (8 patients, 15 keloids) underwent surgical excision alone; Group 2 (10 patients, 19 keloids) received excision plus brachytherapy; Group 3 (9 patients, 14 keloids) received excision plus electron beam radiotherapy. Follow-up evaluations were conducted at 6 months and 2 years.

**Results:**

Surgery combined with radiotherapy showed significantly lower recurrence rates compared to surgery alone, confirming its superior efficacy. No significant difference was observed between brachytherapy and electron beam radiotherapy (*p* = 0.701 for patients, *p* = n/a for keloids), indicating both are effective options. In the subgroup of keloids ≥5 cm, a trend toward lower recurrence was observed with brachytherapy compared to electron beam therapy.

**Conclusions:**

The present study confirms that surgical excision of keloids followed by radiotherapy can be an effective method of keloid treatment. Although no significance was found when comparing the two radiotherapeutic modalities, brachytherapy showed better clinical advantage when treating larger lesion as well as alleviating the symptoms caused by keloids.

## Introduction

Keloids, rather than being skin tumors, are dermal growths related to previous skin trauma or inflammation and they result from abnormal wound healing and tend to recur after excision.^1^

Keloids present clinically as firm and rubbery nodules in an area of prior injury to the skin and frequently project above the underlying skin. They may have a narrow base, resulting in pedunculated lesions, or develop into a more a broad-based plaque. They pervade through the dermis and grow beyond the borders of the original wound. Histologically, keloids exhibit a lack of distinct collagen pattern.^2^, ^3^, ^4^, ^5^

Color ranges from erythematous, flesh-colored, or hyperpigmented and may change as the lesion evolves. Hence, they are aesthetically disfiguring.

About time of formation of keloids may develop as early as 1 to 3 months or as late as one or more years after injury/surgery.^6^

Permanent scars do not occur when the skin injury is about a third of the thickness of the dermis (less than 0.57 mm deep). Conversely, deeper dermal injuries result in permanent scars. Thus, if deep cutaneous wounding disrupts the normal structure of the reticular dermis, keloids form.^7^

They are recognized as a highly fibroproliferative skin disease that usually results from minor injuries. In susceptible individuals, even minor skin injuries such as vaccinations or insect bites, can induce keloid formation.^8^ Despite keloid scars being benign in nature, treatment is challenging because of the high rate of recurrence and the complaints of unbearable pain.^9^

Keloids may have significant cosmetic implications for affected patients as they can grow to be large and disfiguring causing psychosocial issues impairing quality of life. If they are very large in size can lead to functional impairment - movement limitation.^10^, ^11^, ^12^

Due to its diversity of risk factors, clinical appearance, high recurrence rate and lack of proper understanding of the disease, there is presently no gold standard of treatment of progressive keloid lesions.^13^ Current treatments range from corticosteroids applications, surgical techniques, laser therapy, cryotherapy and radiations.

A deeper understanding of the molecular mechanisms that drive keloid development and recurrence would be desirable to open further avenues for the development of innovative treatments.^14^

Among adjuvant treatments, radiotherapy is considered the most effective, significantly reducing recurrence compared to surgery alone.

Initially surgical monotherapy was strongly avoided and used in only extreme cases due to the high rates of recurrence,^15^ which were often much worse than the original lesion.

The high recurrence rate of keloids has led to the use radiation-based treatments to improve cosmetic outcomes after surgery.^16^

Currently, radiotherapy after surgical keloid excision is a recognized the most efficacious modality according to the international advisory panel on scar management and significantly reduces the rate of recurrence.^17^

The reported therapeutic response rates are generally in the range of 67–98%.

However, there is still debate regarding the optimal radiotherapy modality, particularly between brachytherapy and electron beam therapy, with limited direct comparative studies.

The main objective is to compare the efficacy, by means of recurrence rates, between surgical excision alone, electron beam radiotherapy performed immediately after surgical excision, and brachytherapy performed immediately after surgical excision.

## Materials and methods

This is a retrospective observational study conducted on 27 subjects (10 males and 17 females) with overall 48 keloidal scars. Subjects were divided in three groups:

1. Group 1: surgical excision alone;
2. Group 2: surgical excision followed by high-dose-rate brachytherapy (HDR-BT);
3. Group 3: surgical excision followed by electron beam radiotherapy (EBRT).

The following conditions will be considered inclusion criteria (common to the three groups): willingness to participate to the study; female and male on age more than 18 years old; patients who have not undergone radiotherapy; no genetic diseases; patients with one or more keloid (defined as a scar which extends beyond the boundaries of the original skin lesion); patients who have undergone through the same protocol of keloid surgical excision; patients who showed up for the follow-ups.

## Evaluation of treatment

The incidence of the scar recurrence rate was assessed at the 6 month and 2 years after treatment, using the reports of the clinical checks and the evaluation of the photos pre-treatment and during the follow-up visits. Patients are requested to return 2 weeks following the surgery for removing dressing and sutures and disinfecting the scar. Subsequent follow-ups take place at 1 month, 3 months, 6 months and 2 years and beyond after the operation.

Follow up visits are made after therapy with the aim of looking for cases of relapse, questioning patients about any therapy with the aim of looking for cases of relapse, questioning patients about any symptoms, appearance of the scars and whether there are aesthetic problems and limitation in function.

The efficacy of the treatment will be evaluated focusing on three parameters:

1. The subjective scars’ evaluation as reported by a clinician, not affiliated with the work, at the final control in the clinic (2 years).
2. An objective visual comparison of the pre- and post-treatment photographs performed using a dedicated online image overlay tool (Diffchecker.com). (Figure 1A–C).

**Figure 1:**
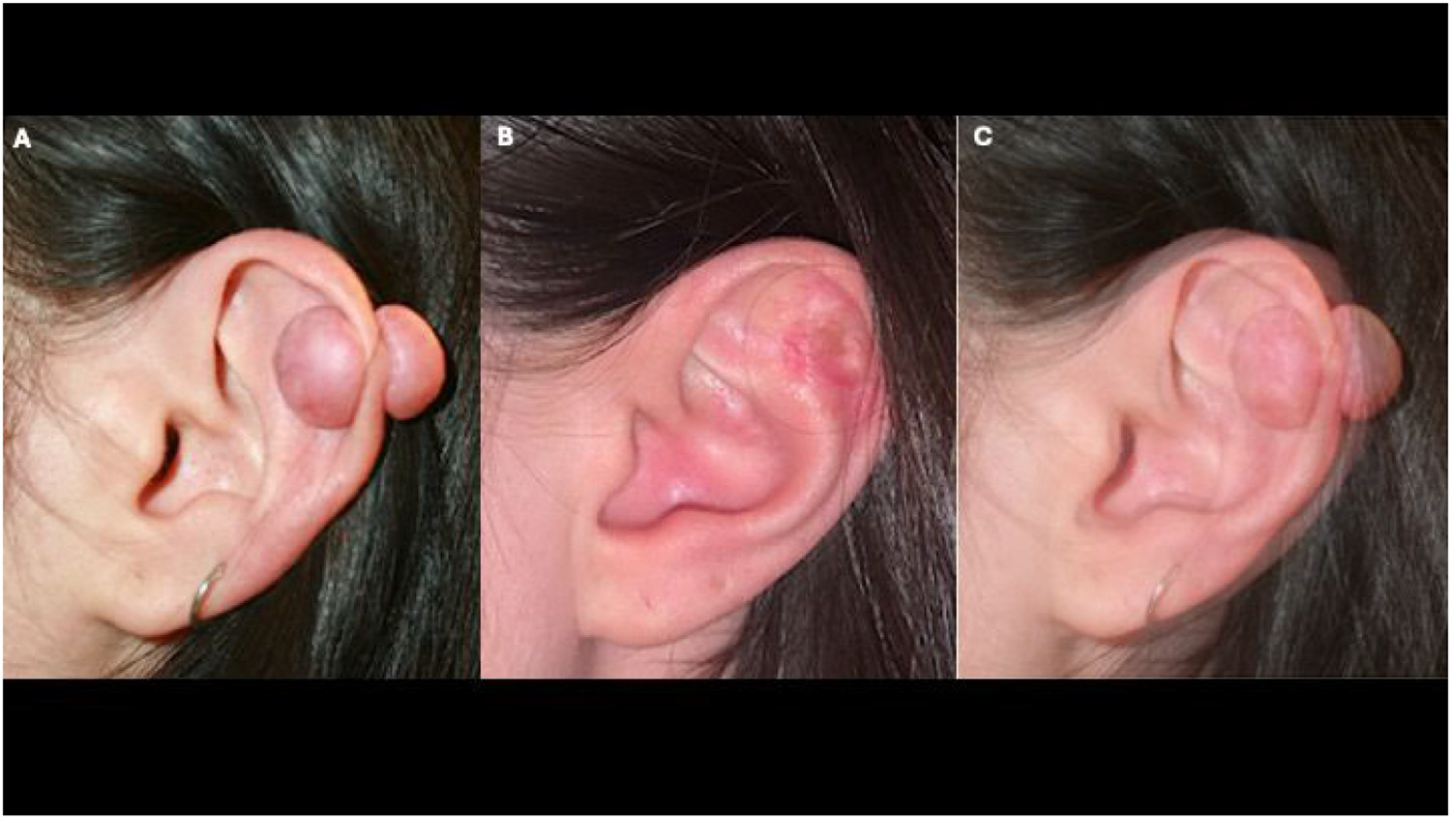
A: Pre-operative photo of a patient with a keloid located on the helix of the left ear, B: Post-operative photo of a patient treated by brachytherapy after surgical excision at the 2 years follow-up, C: Pre and post operative photo comparison through Diffchecker.com software.
3. A particular scale was submitted during the visits. Reported symptoms such as pruritus, pain, hyperesthesia, burning sensation, aesthetic concerns were asked to be evaluated going from 0 to 10, where 0 is the least discomfort and 10 is the maximal discomfort.

## Procedure

### Surgical procedure

The surgical excision intervention is performed for every patient under local or general anesthesia (depending on the scar’s size and number of scars) by single surgeon.

The skin is primarily repaired by tension-reduction suture, consisting of 2 layers of strong suture (deep and superficial fascia layer with monofilament suture composed of polyamide) to approximate the wound edge (wound edge reach to each other after fascia suture).

After surgery, the entire wound is covered with semi occlusive dressing, and the sutures are removed 2 weeks after surgery.

### Radiotherapy procedure

The first dose of postoperative adjuvant radiotherapy is provided within 24 h.^18^^,^^19^

### High-dose-rate brachytherapy protocol

If a subsequent brachytherapy is planned, then a 6-french interstitial plastic catheter is placed during the surgery in the open wound bed (before suturing); a metallic guide is inserted throughout the catheter to avoid its obstruction or deformation during the suturing procedure. HDR-BT was delivered using an Iridium-192 source, for a total dose of 12 Gy in 4 fractions. The first fraction was administered within 90 min from surgery, followed by a second dose 6 h later. Two additional fractions were delivered the following day, also 6 h apart. Radiation was prescribed to a depth of approximately 3–5 mm from the skin surface, corresponding to the dermal fibroblast layer, where the catheter-to-surface distance ranged between 3–10 mm depending on wound thickness. After the final fraction, the catheter was removed and the wound covered with Steri-Strips.

### Electron beam radiotherapy protocol

Treatment was delivered with a linear accelerator using 6–9 MeV electrons, with energy selected according to lesion thickness to ensure adequate coverage. The dose prescription is different according to specific body region:

• 20 Gy in 5 fractions for 5 days (anterior chest wall, scapular region, suprapubic region, and mandible region);
• 15 Gy in 3 fractions for 3 days (other regions);
• 10 Gy in 2 fractions for 2 days to earlobes.^20^

### Statistical analysis

Regarding the statistical analysis of the study: continuous variables were compared using Student’s t-test, categorical variables were compared using the *χ*2 test and Fisher’s exact test, differences between the groups were compared using ANOVA with Bonferroni correction, recurrence over time was evaluated by Kaplan-Meier curve estimates.

## Results

Among the 27 patients (Table 1), a total of 48 keloids were included in this study, distributed as follows: 21 (43.75%) were smaller in size than 5 cm, while 27 (56.25%) were bigger than 5 cm. Specifically, Group 1 included 5 keloids smaller than 5 cm, while 10 keloids bigger than 5 cm. Regarding Group 2 instead, 6 keloids were smaller than 5 cm and 13 keloids were bigger than 5 cm. Finally, Group 3 included 10 keloids smaller than 5 cm and 4 keloids bigger than 5 cm (Table 2).

**Table 1:** Patients demographics.

	Surgery	Brachytherapy	Electron	Total
Number treated	8 (29.6%)	10 (37%)	9 (33.3%)	27
Sex				
Female Male	5 (62.5%) 3 (37.5%)	7 (70%) 3 (30%)	5 (55.5%) 4 (44.4%)	17 (62.9%) 10 (37.1%)
Age	23 ± 3.3	27.8 ± 9.3	27.3 ± 5.8	26 ± 6.9
Ethnicity				
Type 2–3 Type 4 Type 5–6	7 (87.5%) 0 1 (12.5%)	7 (70%) 2 (20%) 1 (10%)	8 (88.8%) 1 (11.1%) 0	22 (81.4%) 3 (11.1%) 2 (7.5%)

Patients demographics in the three groups divided by numbers treated, age, sex and ethnicity.

**Table 2:** Keloid demographics.

	Surgery	Brachytherapy	Electron	Total
Number treated	15 (31.25%)	19 (39.5%)	14 (29.1%)	48
Location				
Ears Back Thorax Shoulders Arms	4 (26.6%) 5 (33.3%) 2 (13.3%) 3 (20%) 1 (6.66%)	8 (42.1%) 5 (26.3%) 3 (15.7%) 2 (10.5%) 1 (5.2%)	3 (21.4%) 3 (21.4%) 1 (7.14%) 6 (42.8%) 1 (7.14%)	15 (31.25%) 13 (27%) 6 (12.5%) 11 (22.9%) 3 (6.25%)
Etiology				
Surgical Piercing Acne Burning Trauma Tatoo Burning and trauma	1 (6.66%) 7 (46.6%) 5 (33.3%) 0 1 (6.66%) 1 (6.66%) 0	6 (31.5%) 6 (31.5%) 5 (26.3%) 0 0 0 2 (10.5%)	4 (28.5%) 2 (14.2%) 5 (35.7%) 3 (21.4%) 0 0 0	11 (22.9%) 15 (31.25%) 15 (31.25%) 3 (6.25%) 1 (2.1%) 1 (2.1%) 2 (4.1%)
Size				
<5 cm >5 cm	5 (33.3%) 10 (66.6%)	6 (31.6%) 13 (68.4%)	10 (71.5%) 4 (28.5%)	21 (43.75%) 27 (56.25%)

Keloids characteristics in the three groups divided by numbers treated, location, etiology and size.

The incidence of the scar recurrence rate was assessed at the 6 month and 2 years after treatment.

At the 6 months breaking point it was found that in Group 1 there was a recurrence of 6 keloids (40%) in 3 patients, 2 of which were smaller than 5 cm while 4 were bigger. In Group 2 there was a recurrence of 2 keloids (10%) in 1 patient, both being bigger than 5 cm. In Group 3 there was no recurrence of keloids.

At the 2 years breaking point, it was found that in Group 1 there was a recurrence of 13 keloids (86%) in 6 patients, 3 keloids being smaller than 5 cm while 10 being bigger than 5 cm. In Group 2 there was a recurrence of 5 keloids (26%) in 3 patients, one keloid being smaller than 5 cm and 4 being bigger than 5 cm. In Group 3 there was a recurrence of 3 keloids (21%) in 2 patients, one keloid being smaller than 5 cm and 2 being bigger than 5 cm.

**Figure 2:**
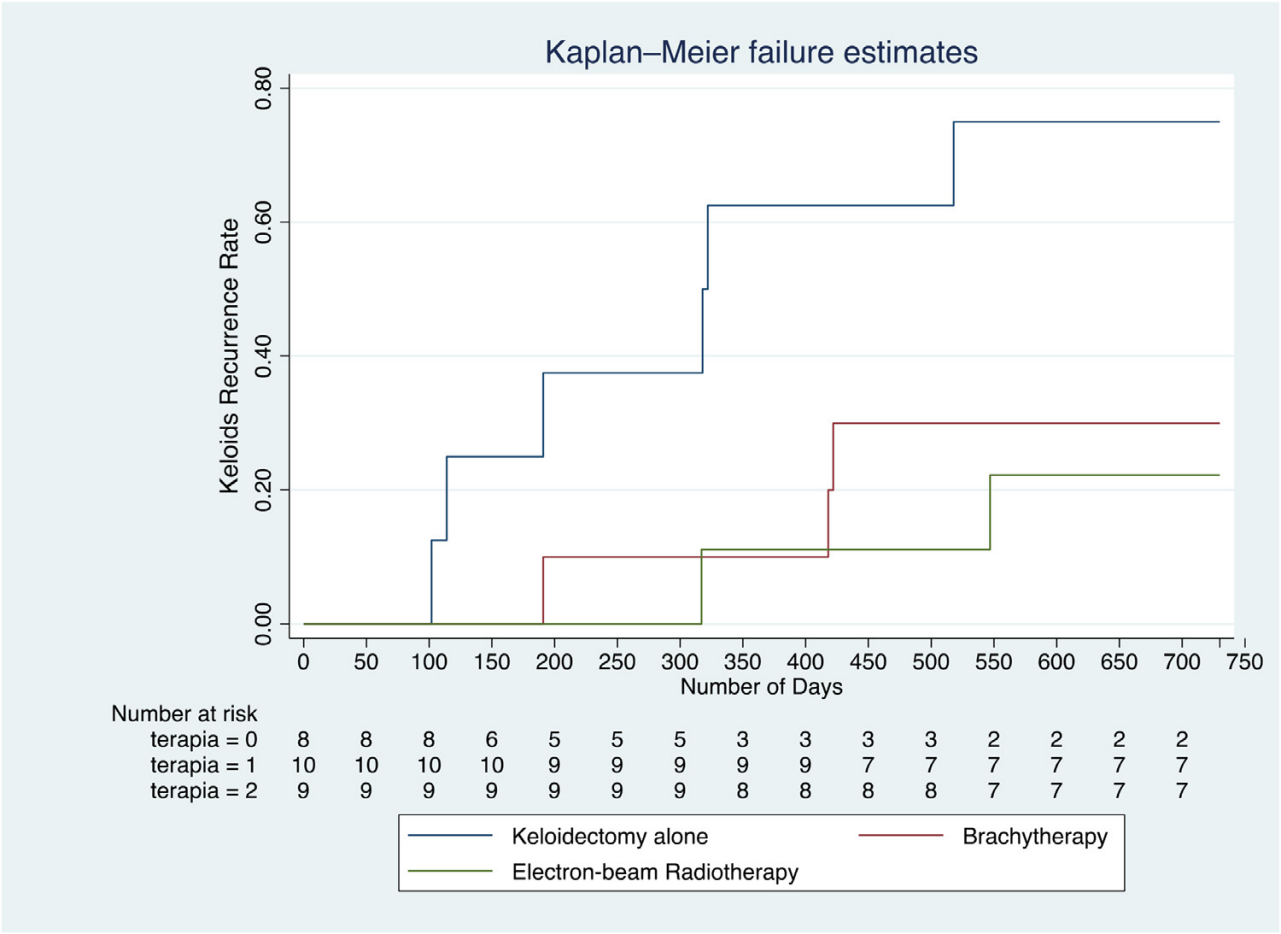
Kaplan-Meier curve failure estimate of therapies. The Kaplan-Meier curve express the overall keloids recurrence rate (y axis) at any giving time expressed in number of days (x axis). Number at risk indicates the patients who haven’t had a keloid recurrence in that specific analysis time (number of days). In the curve the Group 1 (surgery alone) is associated with “terapia = 0”, the Group 2 (surgery and adjuvant brachytherapy) is associated with “terapia = 1” and Group 3 (surgery and adjuvant electron beam) is associated with “terapia = 2”.

In the Kaplan Meier curve is possible to observe the recurrence rate at the first breaking point (6 months) was of 14.81% while the recurrence rate at the second breaking point (2 years) was of 40.74% (Figure 2).

During the follow ups we handed every patient to complete subjectively a scale taking into consideration 5 symptoms being: pruritus, pain, hyperesthesia, burning sensation and aesthetic concern during 4 different timelines (before therapy, after therapy, 6 months, 2 years). At the end of the 2 years follow up we calculated the average mean behind every score handed by the patients.

**Figure 3:**
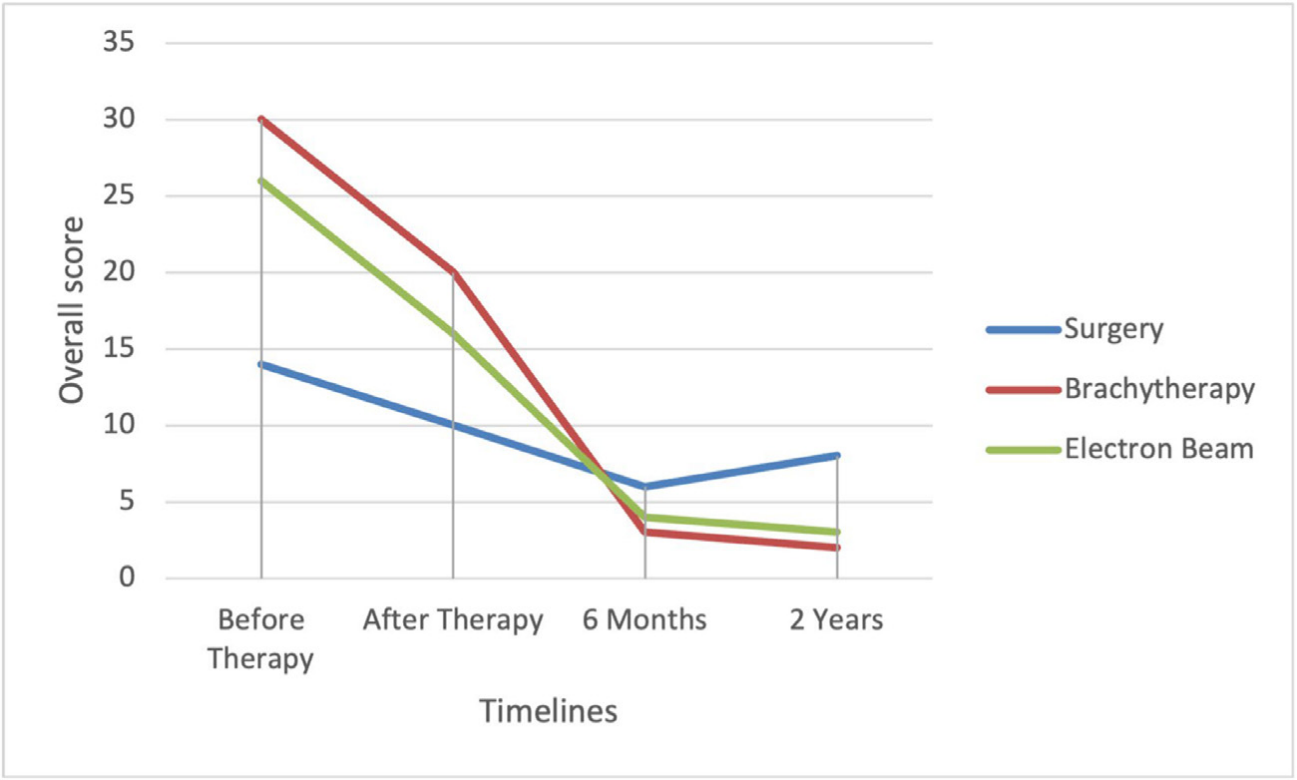
Subjective scale administered to the patients taking in consideration 5 different symptoms The figure expresses the mean score of the evaluated symptoms (y axis) at four different timelines (x axis).

The mean score in Group 1 was of 10,625, where instead in Group 2 and Group 3 is respectively 2.5 and 3 (Figure 3).

The adverse effects of radiotherapy modality can be grouped into acute skin reactions and late complications. Skin damage is a common side effect of radiotherapy, which causes excessive keratosis, skin tissue atrophy, abnormal skin pigments metabolism (pigmentation or depigmentation), and telangiectasia.^21^

The most assessed sides effects of the radiotherapy modality were oedema, erythema and skin pigmentation changes, as similarly seen in the literature^16^ with overall reactions being less than 1%.

## Discussion

Radiotherapy has been shown to be one of the only treatment methods which are effective in preventing keloid scars, particularly in case they were previously excised, with an estimated recurrence rate around 20%. Currently, the most used radiotherapy techniques include external radiotherapy (using superficial X-rays or electrons) and low-dose-rate brachytherapy.^22^ It is noted that the occurrence of late complications increases at doses above 20 Gy.^23^^,^^24^

Our results suggest that irradiating the fresh surgical lesion following surgery would yield favorable results, that is low incidence of recurrences, compared to surgery alone, thus highlighting the effectiveness of surgery in combination with radiotherapy in the treatment of keloid scars. We examined whether EBRT is superior to HDR-BT or vice versa; these results demonstrate that there is no significant statistical difference for the patients recurrences (*p* = 0701) and for the keloids recurrence (*p* = n/a) thus both radiotherapy modalities are efficacious in preventing the recurrence of keloids.

Group 1 proved to be not efficient in preventing the recurrence of keloid scars due to higher rates compared to Group 2 (*p* = 0058) and compared to Group 3 (*p* = 0030) at the 2 years follow-up when analyzing the patient’s recurrence (Table 3). Complementary the same results can be found when taking in consideration the keloids recurrence, with Group 2 (*p* ≤ 0.001) and Group 3 (*p* ≤ 0.001) being the more efficient treatments when compared to Group 1 (Table 4).

**Table 3:** Patients recurrence characteristics.

	Overall (n = 11 (40.7%)	Group 1 (n = 6 (75.0%))	Group 2 (n = 3 (30.0%))	Group 3 (n = 2 (22.2%))	p-value Overall (Group 1 vs Group 2 + 3)	p-value (Group 1 vs Group 2)	p-value (Group 2 vs Group 3)	p-value (Group 1 vs Group 3)
6 months	4 (14.8%)	3 (37.5%)	1 (10.0%)	0 (0%)	**0.031**	0.163	0.330	**0.043**
2 years	11 (40.7%)	6 (75.0%)	3 (30.0%)	2 (22.2%)	**0.018**	**0.058**	0.701	**0.030**

Patients recurrence characteristics with overall recurrence, recurrence in three groups and *p*-value comparison between the groups. Bold numbers are indicating statistical significance.

**Table 4:** Keloids recurrence characteristics.

	Overall (*n* = 21 (43.75%)	Group 1 (*n* = 13 (86.7%))	Group 2 (*n* = 5 (26%))	Group 3 (*n* = 3 (21%))	*p*-value Overall (Group 1 vs Group 2+3)	*p*-value (Group 1 vs Group 2)	*p*-value (Group 2 vs Group 3	*p*-value (Group 1 vs Group 3)
6 months (<5 cm)	2	2 (40.0%)	0 (0%)	0 (0%)	**0.047**	0.181	N/A	0.095
6 months (>5 cm)	6	4 (40.0%)	2 (15.4%)	0 (0%)	0.153	0.341	N/A	0.250
2 years (<5 cm)	5	3 (60.0%)	1 (16.7%)	1 (10%)	0.063	0.242	N/A	0.077
2 years (>5 cm)	16	10 (100%)	4 (30.8%)	2 (40%)	**<0.001**	**<0.001**	0.584	0.066
6 months	8	6 (40%)	2 (10%)	0 (0%)	**0.007**	0.100	0.496	**0.016**
2 years	21	13 (86%)	5 (26%)	3 (21%)	**<0.001**	**<0.001**	n/a	**<0.001**

Keloids recurrence characteristics with overall recurrence, recurrence in the three groups and *p*-value comparison between the groups at 6 and 2 years follow-ups with different lesions size. Bold numbers are indicating statistical significance.

The Kaplan-Meier curve showed how the first recurrence of keloid in a patient not only occurred in Group 1, but it happened at the 100 days point, continuously the recurrence of keloids in Group 1 were more common in the 180 days first breaking point with respect to Group 2 and Group 3. Regarding Group 2 and Group 3, the two curves crossing from 320 days and 420 days show how the two therapies gave the same recurrence rate in the patients at that specific analysis time. However, going through the end of the analysis time we can see a slight lower recurrence rate in Group 3.

Summarizing the results, Group 1 (surgery alone) recurrence rate is constant through the analysis time. Group 2 (HDR-BT) and Group 3 (EBRT) at the first breaking point (180 days) assess the same recurrence rates, while diverging through the second breaking point (720 days).

The total recurrence rate increased between the 6 months and 2 years evaluation, respectively going from 14.81% to 40.74%, demonstrating that perhaps the development of keloids takes place mostly at the 6 months from the moment of lesion.

The subjective scale, evaluating 5 different symptoms, which was supplied to the patients showed us a discrepancy in the results, seeing how the mean score was lower in the patients applied to Group 2 while the patient recurrence rate was higher in this group.

When recurrence rates were not stratified by keloid size (including both lesions smaller than 5 cm and those larger than 5 cm) a clear trend emerged indicating that Group 1 was the least effective treatment, both in the short term (6 months, *p*-value = 0.007) and in the long term (2 years, *p*-value = (*p* ≤ 0.001). Specifically compared to both radiotherapeutical modalities, results show statistical significancy comparing Group 1 vs Group 2 (*p* ≤ 0.001) and comparing Group 1 vs Group 3 (*p* ≤ 0.001) at the 2 years follow-up.

For keloids smaller than 5 cm, both short-term and long-term follow-ups confirmed the superiority of radiotherapy modalities (Groups 2 and 3) over the treatment used in Group 1. The difference was found to be statistically significant at the 6-month follow-up (*p*-value = 0.047), the trend remained consistent over time with an almost significant analysis at the 2 years follow-up (*p*-value = 0.063).

Similarly, in keloids measuring ≥5 cm, radiotherapy-based approaches were again associated with lower recurrence rates. HDR-BT (Group 2) showed a significantly better outcome compared to Group 1 (*p* ≤ 0.001) highlighting its potential effectiveness in treating more extensive lesions.^25^

On the contrary, EBRT (Group 3) when compared to Group 1 was found to not be statistically significant (*p*-value = 0.066).

HDR-BT where keloid fibroblast activity is highest while minimizing deeper-tissue exposure. In contrast, electron beam radiotherapy shows a surface build-up followed by a broader and deeper dose deposition, resulting in a more gradual attenuation. These distinct physical profiles may help explain the slightly better outcomes observed with brachytherapy in larger lesions (≥5 cm), where precise superficial dose confinement is more advantageous.^26^^,^^27^

Regarding safety, in our cohort, toxicity was mild (<1%) and no significant late complications were observed. The rapid dose fall-off of HDR-BT reduces the risk of radiation exposure beyond the target area, whereas electron beam radiotherapy, despite remaining superficial, results in a broader dose distribution within the subcutaneous tissues that may be associated with a slightly higher potential for pigmentary or subdermal effects. Nevertheless, both modalities remain safe, and the doses used for keloids are well below thresholds associated with meaningful long-term risks.

In line with previous findings, including those by Yossi et al., our results confirm the benefit of adjuvant radiotherapy over surgery alone in the treatment of keloids. By incorporating a three-arm comparative design and stratifying lesions by size, this study adds new insights, particularly regarding the potential superiority of HDR-BT in larger keloids (≥5 cm) and refines the understanding of treatment efficacy across different clinical scenarios.

These findings reinforce the idea that radiotherapy modalities should be considered a key component in the multimodal management of keloids, particularly when addressing high-risk cases such as larger lesions or those with a history of recurrence. However, no statistically significant differences were observed between the two radiotherapeutic groups.

Choosing one or another radiation therapy following the keloidectomy can be related to the presence or not of the equipment in the hospital and regarding the costs of the treatments itself. Moreover, HDR-BT cannot be offered to all patients for technical and logistic reasons.^25^ HDR-BT has the highest cost of all the three treatments included in the study, while electron beam therapy comes in second place.

Concluding, although the costs are higher using both radiation treatments after performing a keloidectomy, the higher expenses can be justified by lower recurrency rates and in an overall smaller cost in a long-term treatment.

### Limits of the study

Limits of the study include the following: retrospective clinical study; non-randomized clinical study; small number of patients; nonsystematic allocation of the different keloids localization in the three groups; nonsystematic allocation of the different Fitzpatrick groups.

## Conclusions

The present study suggests that surgical excision of keloids followed by adjuvant radiotherapy can be an effective method of keloid and prevent their recurrence. This combination therapy, using surgical excision followed by radiotherapy appears to be also efficacious in alleviating the symptoms caused by keloids such as pruritus, pain, hyperesthesia, burning and the aesthetic concerns. Recurrence rate comparison between electron beam and brachytherapy was statistically insignificant, both analyzing patients and keloids recurrency rates. Instead, recurrence rate comparison between the keiloidectomy alone and respectively with brachytherapy and with electron beam was found to be statistically significant.

At 2 years of follow-up, an interesting divergence emerged between the two radiotherapy-based treatment modalities when evaluating keloids larger than 5 cm. Although both HDR-BT (Group 2) and EBRT (Group 3) were generally effective in reducing recurrence rates compared to surgery alone (Group 1), HDR-BT demonstrated a superior outcome in this specific subgroup. While the comparison between Group 2 and Group 3 did not reach statistical significance, the numerical difference in recurrence rates suggests a potential clinical advantage for HDR-BT in managing larger and more complex keloids. However, further research is necessary to affirm the efficacy of this form of treatment – in a larger specimen of patients, and perhaps by a randomized controlled trial that would follow the patients for a longer period.

## Funding

None.

## Declaration of competing interest

None declared.

## References

[1] Ogawa R. (2017). Keloid and hypertrophic scars are the result of chronic inflammation in the reticular dermis. Int J Mol Sci.

[2] Gauglitz G.G., Korting H.C., Pavicic T., Ruzicka T., Jeschke M.G. (2011). Hypertrophic scarring and keloids: pathomechanisms and current and emerging treatment strategies. Mol Med.

[3] Al-Attar A., Mess S., Thomassen J.M., Kauffman C.L., Davison S.P. (2006). Keloid pathogenesis and treatment. Plast Reconstr Surg.

[4] Durani P., McGrouther D.A., Ferguson M.W.J. (2009). Current scales for assessing human scarring: a review. J Plast Reconstr Aesthet Surg.

[5] Menashe S., Heller L. (2024). Keloid and hypertrophic scars treatment. Aesthet Plast Surg.

[6] Palko J.R., Arfeen S., Farooq A.V., Reppa C., Harocopos G.J. (2019). Corneal keloid presenting forty years after penetrating injury: case report and literature review. Surv Ophthalmol.

[7] Dunkin C.S.J., Pleat J.M., Gillespie P.H., Tyler M.P.H., Roberts A.H.N., McGrouther D.A. (2007). Scarring occurs at a critical depth of skin injury: precise measurement in a graduated dermal scratch in human volunteers. Plast Reconstr Surg.

[8] An J., Wang G., Xu D., Yin H., Hua Y., Wang C. (2025). Retrospective study on the effectiveness of punch drilling combined with superficial X-ray radiotherapy and intralesional drug injection for keloid treatment. JPRAS Open.

[9] Berman B., Maderal A., Raphael B. (2017). Keloids and hypertrophic scars: pathophysiology, classification, and treatment. Dermatol Surg.

[10] Robles D.T., Berg D. (2007). Abnormal wound healing: keloids. Clin Dermatol.

[11] Maemoto H., Iraha S., Arashiro K., Ishigami K., Ganaha F., Murayama S. (2020). Risk factors of recurrence after postoperative electron beam radiation therapy for keloid: comparison of long-term local control rate. Rep Pract Oncol Radiother.

[12] McGinty S., Keloid S.W.J. (2023).

[13] Nangole F.W., Agak G.W. (2019). Keloid pathophysiology: fibroblast or inflammatory disorders?. JPRAS Open.

[14] Memariani H., Memariani M., Moravvej H., Shahidi-Dadras M. (2021). Emerging and novel therapies for keloids: a compendious review. Sultan Qaboos Univ Med J.

[15] Ogawa R., Dohi T., Tosa M., Aoki M., Akaishi S. (2021). The latest strategy for keloid and hypertrophic scar prevention and treatment: the Nippon Medical School (NMS) protocol. J Nippon Med Sch.

[16] Mankowski P., Kanevsky J., Tomlinson J., Dyachenko A., Luc M. (2017). Optimizing radiotherapy for keloids: a meta-analysis systematic review comparing recurrence rates between different radiation modalities. Ann Plast Surg.

[17] Zainib M., Amin N.P. (2023). StatPearls.

[18] Yamawaki S., Naitoh M., Ishiko T., Muneuchi G., Suzuki S. (2011). Keloids can be forced into remission with surgical excision and radiation, followed by adjuvant therapy. Ann Plast Surg.

[19] Peng Q., Lu Y., Huang R., Chen R. (2025). Should we do postoperative radiotherapy after keloid excision as soon as possible? A systematic review and meta-analysis. Aesthetic Plast Surg.

[20] Ogawa R., Miyashita T., Hyakusoku H., Akaishi S., Kuribayashi S., Tateno A. (2007). Postoperative radiation protocol for keloids and hypertrophic scars: statistical analysis of 370 sites followed for over 18 months. Ann Plast Surg.

[21] Tsai S-L, Tsai Y-C, Weng Y-T, Huang W-Y, Wang C-H (2025). Keloid excision with primary closure combined with superficial radiation therapy (SRT-100). Ann Plast Surg.

[22] Ogawa R., Mitsuhashi K., Hyakusoku H., Miyashita T. (2003). Postoperative electron-beam irradiation therapy for keloids and hypertrophic scars: retrospective study of 147 cases followed for more than 18 months. Plast Reconstr Surg.

[23] Sakamoto T., Oya N., Shibuya K., Nagata Y., Hiraoka M. (2009). Dose-response relationship and dose optimization in radiotherapy of postoperative keloids. Radiother Oncol.

[24] Ogawa R., Tosa M., Dohi T., Akaishi S., Kuribayashi S. (2019). Surgical excision and postoperative radiotherapy for keloids. Scars Burn Heal.

[25] Yossi S., Krhili S., Mesgouez-Nebout N. (2013). Traitement postopératoire des cicatrices chéloïdes : électrons ou irradiation interstitielle ? [Adjuvant treatment of keloid scars: electrons or brachytherapy?]. Cancer Radiother.

[26] Guix B., Henríquez I., Andrés A., Finestres F., Tello J.I., Martínez A. (2001). Treatment of keloids by high-dose-rate brachytherapy: a seven-year study. Int J Radiat Oncol Biol Phys.

[27] van Leeuwen M.C., Stokmans S.C., Bulstra A.E. (2015). Surgical excision with adjuvant irradiation for treatment of keloid scars: a systematic review. Plast Reconstr Surg Glob Open.

